# Characterization of a λ-Carrageenase Mutant with the Generation of Long-Chain λ-Neocarrageenan Oligosaccharides

**DOI:** 10.3390/foods13121923

**Published:** 2024-06-18

**Authors:** Zewei Lu, Hong Jiang, Dianqi Yang, Hengxin Tang, Hamed I. Hamouda, Tao Wang, Xiangzhao Mao

**Affiliations:** 1 State Key Laboratory of Marine Food Processing and Safety Control, College of Food Science and Engineering, Ocean University of China, Qingdao 266404, China; 2 Laboratory for Marine Drugs and Bioproducts of Qingdao National Laboratory for Marine Science and Technology, Qingdao 266237, China; 3 Qingdao Key Laboratory of Food Biotechnology, Qingdao 266404, China; 4 Key Laboratory of Biological Processing of Aquatic Products, China National Light Industry, Qingdao 266404, China; 5 Sanya Ocean Institute, Ocean University of China, Sanya 572024, China; 6 Department of Food Science and Technology, School of Agriculture and Biology, Shanghai Jiao Tong University, Shanghai 200240, China

**Keywords:** λ-carrageenan oligosaccharides, λ-carrageenase, pyrroloquinoline quinone-like domain, expression, enzymatic properties

## Abstract

λ-carrageenan oligosaccharides can be widely applied in the food, pharmaceutical, medicine and cosmetic industries due to their abundant bioactivities, and they are important products for the high-value utilization of λ-carrageenan. However, oligosaccharides with different degrees of polymerization have different properties, and the final products of λ-carrageenase reported so far are mainly λ-neocarrabiose, λ-neocarratetraose and λ-neocarrahexaose without longer-chain oligosaccharides. Further research is consequently required. Herein, a mutant λ-carrageenase was constructed by deleting the pyrroloquinoline quinone-like domain of OUC-CglA derived from *Maribacter vaceletii*. Interestingly, it was discovered that the majority of final products of the mutant OUC-CglA-DPQQ were long-chain oligosaccharides with a polymerization degree of 10–20, which underwent significant changes compared to that of OUC-CglA. Additionally, without the pyrroloquinoline quinone-like domain, fewer inclusion bodies were produced throughout the expression process, and the yield of the λ-carrageenase increased about five-fold. However, compared to its parental enzyme, significant changes were made to its enzymatic properties. Its optimal temperature and pH were 15 °C and pH 7.0, and its specific activity was 51.59 U/mg. The stability of the enzyme decreased. Thus, it was found that the deleting domain was related to the formation of inclusion bodies, the stability of the enzyme, the activity of the enzyme and the composition of the products.

## 1. Introduction

Carrageenan, as a polysaccharide containing a large number of sulfate groups, performs a range of useful functions, such as strong gum-forming capabilities, chemical stability and excellent bioactivities, and it is used in a wide range of applications in the food, beverage and cosmetic industries [1]. However, as a polymeric linear polysaccharide, the high viscosity, low solubility, and bioavailability limit the further exploitation of carrageenan [2,3].

Carrageenan oligosaccharides, which are produced from the degradation of carrageenan, have not only a variety of biological activities, such as anti-inflammatory, anti-coagulant, anti-tumor and anti-thrombotic activities, but also have good solubility and bioavailability [4,5,6,7].

It is known from the structure of carrageenan that λ-carrageenan oligosaccharides have a larger amount of sulfate groups than other carrageenan oligosaccharides, and research has revealed that the degree of polymerization significantly affects the biological activity and range of applications of carrageenan oligosaccharides [8]. For example, low-molecular-mass carrageenan oligosaccharides showed better anti-tumor activity [9]; λ-carrageenan oligosaccharides with a molecular mass of 5.9 kDa had lower anti-coagulant activity, but they had a significant inhibitory effect on the spread of MDA-MB-231 breast cancer cells [10]; and new ι-carrageenan tetrasaccharides not only had the function of alleviating lipid metabolism disorders but also had a significant inhibitory effect on pancreatic β-cell apoptosis4 [11,12]. Long-chain oligosaccharides, with a higher degree of polymerization, have some biological properties that short-chain oligosaccharides do not. For instance, long-chain chitin oligosaccharides have long been known as plant elicitors, and they are recognized as microbe-associated molecular patterns and trigger immunity signaling in several species [13]. A mixture of short-chain galacto-oligosaccharides and long-chain fructo-oligosaccharides supplementation could reduce the cumulative incidence of atopic dermatitis in high-risk infants [14], while alginate oligosaccharides with a polymerization degree of 6.8 showed the best promotion of plant growth and significant inhibition of *Pseudomonas aeruginosa* [15]. Odd-numbered agarooligosaccharides with a high polymerization degree expressed the highest activity by scavenging intracellular oxidative damage to protect cells from reactive oxygen species-induced death [16]. Consequently, long-chain oligosaccharides have attracted a lot of research interest.

Some progress has been made in the enzymatic degradation of λ-carrageenan. Several λ-carrageenases have been found and characterized, and the ultimate products of these λ-carrageenases which have been reported until now, mostly consist of λ-neocarrabiose (Nλ2), λ-neocarratetraose (Nλ4) and λ-neocarrahexaose (Nλ6), and they are all short-chain oligosaccharides [17,18,19,20,21,22]. Therefore, more research studies into the preparation of long-chain λ-neocarrageenan oligosaccharides are essential.

In our previous study, a λ-carrageenase OUC-CglA from the marine bacterium *M. vaceletii* belonging to the glycoside hydrolase family 150 (GH150) was identified and characterized [20]. Notably, OUC-CglA, with a maximum specific activity of up to 418.68 U/mg, is a low-temperature-adapted enzyme whose product is mainly composed of short-chain oligosaccharides [20]. It was found that OUC-CglA has a pyrroloquinoline quinone (PQQ)-like helical folding region and its function has yet to be explored [20].

PQQ is an aromatic-reducing quinone, the third class of redox cofactors in addition to the well-known cofactors nicotinamide (NAD(P)^+^) and flavin (FAD, FMN) [23]. PQQ has several functional properties. For example, free PQQ is currently found in various foods and milk [24,25]. It is not only an important nutrient for mouse growth and development but also has the function of protecting living cells and active substances from oxidation [26,27,28]. Moreover, PQQ has the function of delaying skin aging caused by ultraviolet light and can inhibit cholesterol levels in serum [29,30]. In addition, it has interactions with Ca^2+^ and other metal ions [31]. However, the PQQ-like domain, not like the free PQQ, contains several β-strains and forms so-called β-barrel proteins, which were reported to be essential components of the outer membrane of mitochondria, chloroplasts and Gram-negative bacteria, and they form active transporters, pores, enzymes and structural proteins [32,33]. The PQQ-like domain of the OUC-CglA is thus thought to have a similar redox function and a possible function in stabilizing the protein structure and forming catalytic grooves, as above [32]. 

In this study, the PQQ-like domain was excised and its effects were explored. It was discovered that the majority of final products of the mutant OUC-CglA-DPQQ were long-chain oligosaccharides, which had a difference compared to that of other λ-carrageenases reported [17,18,19,20,21,22], suggesting that the mutant could be a candidate enzyme to produce longer-chain λ-neocarrageenan oligosaccharides. Additionally, without the PQQ-like domain, fewer inclusion bodies were produced throughout the expression process and the yield of the λ-carrageenase increased about five-fold. Moreover, the enzymatic properties of the mutant OUC-CglA-DPQQ were compared to its parental enzyme.

## 2. Materials and Methods

### 2.1. Materials and Microbial Strains

The λ-carrageenan utilized to determine the activity was bought from Shanghai TCI-SCT (Shanghai, China). The agarose, κ- and ι-carrageenan and were purchased from Sigma (St. Louis, MO, USA). The porphyran used for the substrate specificity determination was extracted in the same way as in the previous research [20,34,35]. the *Escherichia coli* DH5α was acquired from Tsingke Biotechnology Co., Ltd. (Beijing, China) and served as the cellular cloning host. *E. coli* BL21 (DE3) (Tsingke Biotech, Beijing, China) was used as the expression vector. The λ-carrageenan substrates used in this research, if not specifically specified, all had a concentration and pH of 3 mg/mL and pH 7.0.

### 2.2. Domain Analyses and Cloning of Linearized DNA

**Figure 1:**
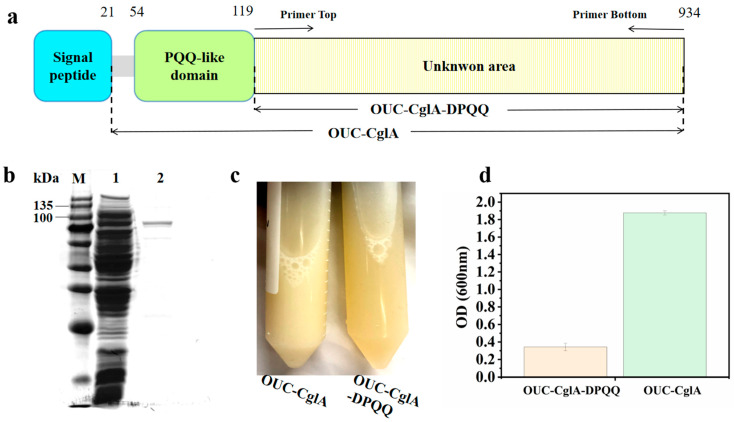
Schematics indicating the primer sets used to amplify the mutant λ-carrageenase OUC-CglA-DPQQ with the PQQ-like domain removed (**a**) and the expression and purification of the mutant λ-carrageenase OUC-CglA-DPQQ. (**b**) SDS-PAGE analysis of the purified OUC-CglA-DPQQ. Lane M, protein molecular mass marker. Lane 1, the crude enzyme extract. Lane 2, OUC-CglA-DPQQ purified by Ni-affinity chromatography. (**c**) Comparison of the cell disruption liquid clarity of the mutant λ-carrageenase OUC-CglA-DPQQ and OUC-CglA with their OD600 (**d**).

The DNA sequence of OUC-CglA from *M. vaceletii* was amplified from previous research [20]. The conserved domains of the enzyme were analyzed by the domain analysis website (https://www.genome.jp/tools/motif/, (accessed on 21 March 2022)). In order to elucidate the effect of the PQQ-like domain on the enzyme activity and degradation pattern, Primer-Top (AGATATACCATGCGCGAAAACAACGCGCCA) and Primer-Bottom (GCGTTGTTTTCGCGCATGGTATATCTCCTTC) were designed and synthesized by Tsingke Biotechnology Co., Ltd. (Beijing, China) to obtain the linearized DNA of the mutant OUC-CglA-DPQQ with amino acids from 1 to 119 of the N-terminal side removed, including the signal peptide and the PQQ-like domain of the λ-carrageenase OUC-CglA. The primers and corresponding amplified DNA fragments are shown in Figure 1, and the PCR amplification was performed in a thermal cycler under the following conditions: 95 °C for 5 min; 98 °C for 10 s, 55 °C for 15 s, 72 °C for 8 min for 30 cycles; and 72 °C for 10 min.

### 2.3. Recombinant Plasmid Construction and Purification of the Enzyme

With Primer-Top and Primer-Bottom, the linearized DNA of the mutant OUC-CglA-DPQQ was amplified using the full gene sequence of OUC-CglA as the template and the circular DNA was obtained by ligating it with the ClonExpress^®^ Ultra One Step Cloning Kit (Vazyme, Nanjing, China), and then the circularized DNA was transformed into *E. coli* DH5α receptor cells to obtain the recombinant plasmid carrying the truncated λ-carrageenase gene *OUC-CglA-DPQQ* with the C-terminal His tag, which was verified by sequencing and then transferred into *E. coli* BL21(DE3) for induced expression.

The OUC-CglA-DPQQ expression was induced by ZYP-5052 medium with a volume of 50 mL. It was cultured at 20 °C for 42–48 h. After derivation, the bacteria were obtained by centrifugation at 10,000× *g* for 15 min. The sedimentation was resuspended with 50 mM potassium phosphate buffer (pH 7.0) and subsequently fractured by ultrasonic disruption for 25 min. Finally, the crude enzyme solution was obtained by centrifugation at 10,000× *g* for 15 min at 4 °C.

The purification of the enzyme was performed using an Ni-NTA resin packing column. The purified protein was then detected by sodium dodecyl sulfate-polyacrylamide gel electrophoresis (SDS-PAGE), and the concentration of OUC-CglA-DPQQ was quantified by a Bradford protein assay [36].

### 2.4. Assay of Enzyme Activity

The reaction system for measuring the specific activity of OUC-CglA-DPQQ was composed of 40 μL purified enzyme and 100 μL substrate. After incubation at its optimal temperature for 15 min, the reaction was stopped by heating for 5 min in a boiling water bath. The amount of the released reducing sugar was measured by the 3,5-dinitrosalicylic acid (DNS) assay. One unit (U) of enzyme activity was defined as the amount of enzyme that produced 1 μmol of reducing sugar under the optimized conditions.

### 2.5. Bioinformatics Analysis, Structure Model Prediction and Molecular Docking

The PQQ-like domain and other PQQ-containing quinoproteins with known functions obtained from the NCBI database was collected. Then, phylogenetic tree analysis was performed using MEGA version 7.0 to explore the possible functions of this domain. The three-dimensional (3D) structure model of the mutant OUC-CglA-DPQQ was predicted by the way described in a previous study using AlphaFold2 (https://colab.research.google.com/github/sokrypton/ColabFold/blob/main/AlphaFold2.ipynb, (accessed on 15 March 2022)) [20], and the highest structural confidence was selected as the template [37]. Molecular docking with different λ-carrageenan oligosaccharides, including Nλ6 and λ-neocarraoctasaccharide (Nλ8), was performed using AutoDock Vina 1.1.2 [38,39]. The analysis of the geometric conformation, distance of the amino acids and hydrogen-bonding interactions of the protein models was performed using Pymol 2.4.0. In addition, the size of the binding pocket of the protein OUC-CglA-DPQQ and OUC-CglA was predicted using the online website DoGSiteScorer (https://proteins.plus/, (accessed on 10 July 2022)) to explore the possible reasons for the change in the final products.

### 2.6. Function of PQQ-like Domain in Inclusion Body Formation

The crushed solution of OUC-CglA and OUC-CglA-DPQQ after cell-breaking was centrifuged at 10,000× *g* for 15 min at 4 °C, the precipitate was discarded, the volume of supernatant was measured, and the protein content of the supernatant was determined by a Bradford protein assay. The absorbance of 200 μL of the supernatant was measured at 600 nm to identify the clarity of the cell disruption liquid, and the total amount of pure enzyme expressed was determined after purifying by an Ni-NTA column.

### 2.7. Characterization of the Purified OUC-CglA-DPQQ

The optimal temperature for the mutant of hydrolyzing λ-carrageenan was determined by mixing 40 μL of the purified enzyme and 100 μL of the λ-carrageenan in the water bath for 15 min, whose temperatures ranged from 10 to 40 °C. Similarly, the optimum pH was performed, respectively, in 50 mM sodium citrate buffer (pH 4.0 to 6.0), 50 mM potassium phosphate buffer (pH 6.0 to 8.0), and 50 mM Tris-HCl buffer (pH 8.0 to 9.0) at 15 °C in the water bath for 15 min. The thermal stability of OUC-CglA-DPQQ was determined by incubating the pure enzyme at each temperature separately from 4 to 30 °C, and then the residual enzyme activity was tested after various incubating times. Similarly, the pH stability of the purified OUC-CglA-DPQQ was tested after storing it in the buffers mentioned above (pH 5.0 to 8.0) at 4 °C for 8 h.

To assess the resistance of the mutant to metal ions and chemicals, a series of metal ions (Na^+^, K^+^, Cu^2+^, Mg^2+^, Zn^2+^, and Fe^3+^) and chemicals (SDS and Na_2_EDTA) were separately applied to analyze their influence on the enzyme activity with a terminal concentration of 1 and 10 mM. The reaction consisting of 100 μL λ-carrageenan and 40 μL purified enzyme was processed at 15 °C for 15 min.

The hydrolyzing activity of the mutant OUC-CglA-DPQQ against κ-carrageenan, ι-carrageenan, agarose and porphyran was examined, and its substrate selectivity was elucidated. The reaction system was composed of 40 μL purified enzyme and 100 μL different substrate solutions (3 mg/mL, pH 7.0). After incubation at 15 °C for 60 min, the reaction was stopped by being boiled in the water bath for 5 min and its activity was measured.

The results provided in this work were the average value of triplicate independent experiments. The two-tailed unpaired *t* test was performed by IBM SPSS Statistics 26 (SPSS Inc., Chicago, IL, USA).

### 2.8. Products’ Analyses of OUC-CglA-DPQQ

To analyze the ultimate product after a thorough reaction, an excess of 5 U of the enzyme was added to 100 μL λ-carrageenan substrate and incubated for 72 h at 15 °C. Then, scanning electron microscopy (SEM) and high-performance liquid chromatography-mass spectrometry (HPLC-MS) were performed to analyze the samples. The detailed methods and the parameters of the SEM and HPLC-MS are the same as in a previous study [20].

### 2.9. Hydrolysis Process Analysis

It was performed by incubating 1.5 U of the purified enzyme with 100 μL λ-carrageenan at 20 °C separately for 2, 4, 8, 16, 24, 36 and 48 h. The reaction was stopped by being boiled in the water bath for 5 min. The samples after boiling were then centrifuged at 10,000× *g* for 10 min at 4 °C and filtered using a 0.45 μm water-based filter membrane. In order to clarify the depolymerization pattern and hydrolysis process of OUC-CglA-DPQQ, analysis of the 100 μL products at different incubation times was performed by an HPLC system equipped with a Superdex peptide 10/300 GL column and RID. 

## 3. Results and Discussion

### 3.1. Sequence Analysis of λ-Carrageenase Mutant OUC-CglA-DPQQ

**Figure 2:**
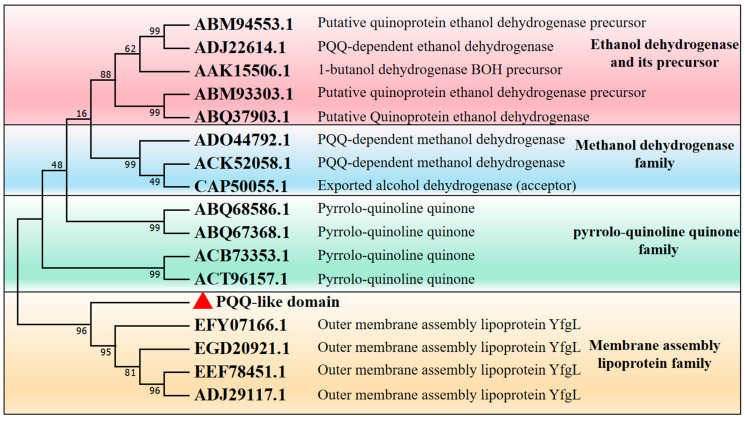
Phylogenetic analysis of the PQQ-like domain and other subunit composition of PQQ-containing quinoproteins from the NCBI database and their functions were marked behind the protein accession numbers. PQQ-like domain was marked in red.

OUC-CglA’s sequencing analysis indicated that the protein’s structure consists of a signal peptide, a PQQ-like domain, and an unidentified functional region. To investigate the effect of the PQQ-like domain on the hydrolyzing performance of the enzyme and its specific activity, the Primer-Top and Primer-Bottom were designed to construct the λ-carrageenase mutant OUC-CglA-DPQQ deleting the PQQ-like domain. The gene encoding the mutant λ-carrageenase OUC-CglA-DPQQ was obtained from the OUC-CglA plasmid by PCR amplification, and it was composed of 2445 nucleotide bases. The nucleotide bases encoded 815 amino acid residues without the PQQ-like domain (Figure 1a). The phylogenetic analysis of the PQQ-like domain and other PQQ-containing quinoproteins with known functions obtained from the NCBI database revealed that the PQQ-like domain does not belong to the pyrrolo-quinoline quinone (PQQ) family, instead, it belongs to the membrane assembly lipoprotein family, which was reported to have the function of stabilizing the protein structure and forming catalytic grooves (Figure 2) [31]. Thus, it can be implied that the PQQ-like domain’s function was more similar to that of the membrane assembly lipoprotein.

### 3.2. Heterologous Expression and Purification of OUC-CglA-DPQQ

The verification of the crude enzyme activity obtained from the fermentation revealed that the mutant was successfully produced. The molecular mass of the purified enzyme was approximately 93.7 kDa according to the protein band (Figure 1b), whereas that of OUC-CglA was 105.1 kDa [20]. It exhibited a purification fold of 2.28 and a recovery yield of 9% (Table S1). The specific activity of the purified enzyme was 51.59 U/mg, while the activity of the parental enzyme was determined to be 418.68 U/mg, which was 19% of the OUC-CglA (Table S1) [20]. As mentioned before, the PQQ-like domain has the ability to form active transporters, pores, enzymes and structural proteins [32]. The absence of the PQQ-like domain is likely to have reduced the enzyme activity, and it also affected the enzyme’s stability and size of the catalytic groove. 

During the purification process, the clarity of the cell disruption liquid of OUC-CglA-DPQQ was found to be higher than that of OUC-CglA (Figure 1c), with an OD600 of 0.343 compared to the higher OD600 of the OUC-CglA cell disruption liquid of 1.877 (Figure 1d), indicating that the cell disruption liquid of the mutant λ-carrageenase OUC-CglA-DPQQ contained fewer inclusion bodies. The total protein and purified pure enzyme protein content of the cell disruption liquid were measured, and the results showed that the total protein content of the mutant cell disruption liquid was 84.438 mg, which was significantly higher than that of OUC-CglA at 59.248 mg. The pure enzyme protein content of OUC-CglA-DPQQ was 3.28 mg (4% of total protein content), which was about 5-fold that of OUC-CglA at 0.68 mg (1% of total protein content) (Table 1). 

This indicated that the PQQ-like structural domain is also related to the formation of inclusion bodies. And since it was a member of the membrane assembly lipoprotein family, as the phylogenetic tree in Figure 2 showed, the significant increase in the content of the protein in the cell disruption liquid was probably due to its losing the function of binding to the cell membrane [32].

### 3.3. Characteristics of OUC-CglA-DPQQ

**Figure 3:**
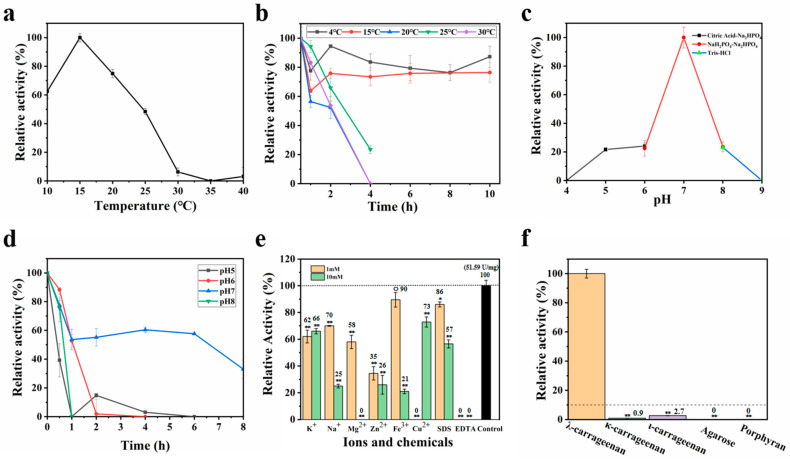
Biochemical characteristics of the purified OUC-CglA-DPQQ. Effects of different temperatures on the λ-carrageenan hydrolysis activity (**a**) and stability (**b**) of the purified OUC-CglA-DPQQ. Effects of different pHs on the λ-carrageenan hydrolysis activity (**c**) and stability (**d**) of the purified OUC-CglA-DPQQ. (**e**) Effects of a series of metal ions and chemicals with 1 mM and 10 mM concentrations on the λ-carrageenan hydrolysis activity of the purified OUC-CglA-DPQQ. (**f**) Relative activity of OUC-CglA-DPQQ against different red algae-derived polysaccharides, including λ-carrageenan, ι-carrageenan, κ-carrageenan, agarose, and porphyran. Significance analysis results are shown in the figure: ○ *p* > 0.05 = no difference; * *p* < 0.05 = difference; ** *p* < 0.01 = significant difference.

The mutant OUC-CglA-DPQQ, which had the PQQ-like domain removed, had an optimal temperature of 15 °C (Figure 3a), making it a lower-temperature-suited enzyme than OUC-CglA. However, the λ-carrageenases from *Pseudoalteromonas carrageenovora* and *Wenyingzhuangia aestuarii* performed better when it was 35 °C and 30 °C, while the Cga-L50 from *Bacillus* sp. had a better activity at 75 °C [17,18,20,22]. Its temperature stability had decreased dramatically compared to OUC-CglA, but it was more stable at 15 °C and 10 °C [20]. It was obvious that the stability of the mutant was better under lower-temperature conditions, but its activity largely disappeared after incubation above 20 °C for more than 4 h (Figure 3b), while OUC-CglA only showed a significant decrease in activity after incubation for more than 6 h above 30 °C [20], proving that the PQQ-like domain may have an impact on the enzyme’s thermal stability. OUC-CglA-DPQQ’s optimal pH was almost consistent with the parental enzyme OUC-CglA (pH 7.0), and the optimal pH of the λ-carrageenases from *P. carrageenovora* and *Pseudoalteromonas* sp. CL19 was 7.5 and 7.0, while the Cga-L50 from *Bacillus* sp. and the Cgl150A_Wa from *Wenyingzhuangia aestuarii* had an optimal pH of 8.0 [17,18,20,21,22]. Below pH 6.0 and above pH 8.0, the activity significantly decreased and was less than 20% of that at pH 7.0 (Figure 3c). Its pH stability also became worse compared to OUC-CglA [20]. OUC-CglA-DPQQ’s activity reduced dramatically after 1 h of incubation in various buffers of different pH, but only to less than 50% of its initial value after 6 h at pH 7.0 (Figure 3d). In contrast, the activity of OUC-CglA started to decline significantly after 5 h incubation in buffers of varying pH [20]. This suggested that the PQQ-like domain also had an effect on the pH stability of the enzyme.

It was found that metal ions such as Zn^2+^ and Fe^3+^, which originally promoted the hydrolyzing activity of OUC-CglA [20], became inhibitors of OUC-CglA-DPQQ, while other metal ions such as K^+^, Na^+^, Mg^2+^, Cu^2+^ and the chemical reagents SDS and EDTA inhibited the enzyme activity (Figure 3e), corresponding to PQQ’s previously mentioned function of binding to metal ions, without which the enzyme cannot be activated by metal ions [31].

Taking the activity of the mutant OUC-CglA-DPQQ against λ-carrageenan as 100%, the relative activity of the mutant against κ-carrageenan was 0.9% and that for ι-carrageenan was 2.7%, while no significant activity was observed for agarose and porphyran, demonstrating that the OUC-CglA-DPQQ’s substrate selectivity has improved since being modified (Figure 3f) [20].

### 3.4. Product Analysis

**Figure 4:**
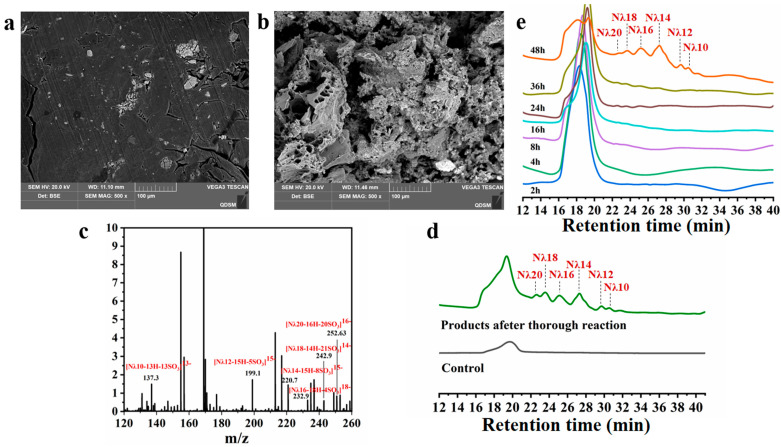
Analyses of the degradation products of OUC-CglA-DPQQ. SEM images of λ-carrageenan (**a**) and λ-carrageenan treated after OUC-CglA-DPQQ (**b**). MS spectrum (**c**) and HPLC (**d**) of the products harvested after a sufficient reaction. HPLC chromatogram of the products of OUC-CglA-DPQQ against λ-carrageenan changes over time (**e**).

The microstructures of the OUC-CglA-DPQQ-treated λ-carrageenan and λ-carrageenan were observed by scanning electron microscopy. As mentioned before, the surface of the λ-carrageenan untreated by λ-carrageenase was smooth (Figure 4a), while the surface of the OUC-CglA-treated product was rough and porous, which indicated that the substrate was disrupted and further hydrolyzed by OUC-CglA [20]. Herein, the surface of the OUC-CglA-DPQQ-treated product was more uneven and had a looser structure compared to that of the OUC-CglA-treated product (Figure 4b), which indicated that the degree of polymerization of the product had changed [20]. The product identification by HPLC-MS revealed that the final products of the mutant were mostly long-chain oligosaccharides, including λ-neocarradecasaccharide (Nλ10), λ-neocarradodecasaccharide (Nλ12), λ-neocarratetradecasaccharide (Nλ14), λ-neocarrahexadecasaccharide (Nλ16), λ-neocarraoctadecasaccharide (Nλ18), and λ-neocarraeicosaccharide (Nλ20) (Figure 4c,d), while those of OUC-CglA were mainly oligosaccharides with lower polymerization degree such as Nλ2, Nλ4 and Nλ6 [20]. The ultimate products of the reported λ-carrageenases are currently mostly Nλ2, Nλ4 and Nλ6 [17,18,19,20,21,22]. Nevertheless, the ultimate products of the mutant λ-carrageenase OUC-CglA-DPQQ without the PQQ-like domain were notably distinct from those produced by all the other λ-carrageenases described, revealing it to be a promising tool for the preparation of long-chain oligosaccharides and suggesting that the PQQ-like domain may be part of the catalytic groove and related to the minimal substrate that the groove can bind [32].

### 3.5. Hydrolysis Process Analysis

The samples of different reaction times were observed by HPLC. Specifically, the long-chain oligosaccharides started to appear when the reaction proceeded to 24–36 h. As the reaction proceeded to 48 h, shorter-chain oligosaccharides of Nλ10, Nλ12, Nλ14 and Nλ16 began to accumulate (Figure 4e), indicating that OUC-CglA-DPQQ was also a typical endo-type λ-carrageenase capable of randomly recognizing and hydrolyzing the β-1,4 glycosidic bonds of λ-carrageenan. In contrast, long-chain oligosaccharides started to appear after 4 h of the OUC-CglA reaction, and short-chain oligosaccharides of Nλ2, Nλ4 and Nλ6 started to accumulate after 10 h of the reaction [20], further suggesting that the PQQ-like domain has an effect on the final products of the enzyme and may be related to the binding of the substrate and catalytic groove [32]. In general, the degradation process of other endo-type carrageenases, such as κ-carrageenaes and ι-carrageenaes, began with the appearance of high-molecular-mass products and ended with the accumulation of low-molecular-mass products [40,41,42]. It is hypothesized that OUC-CglA-DPQQ’s hydrolysis mechanism differs from that of traditional carrageenases. The investigation of their degradation patterns, particularly the connection between their sequences, structures, and degradation patterns, was not well established since the studies on λ-carrageenase are few.

### 3.6. Analysis of Catalytic Centre and Molecular Docking

**Figure 5:**
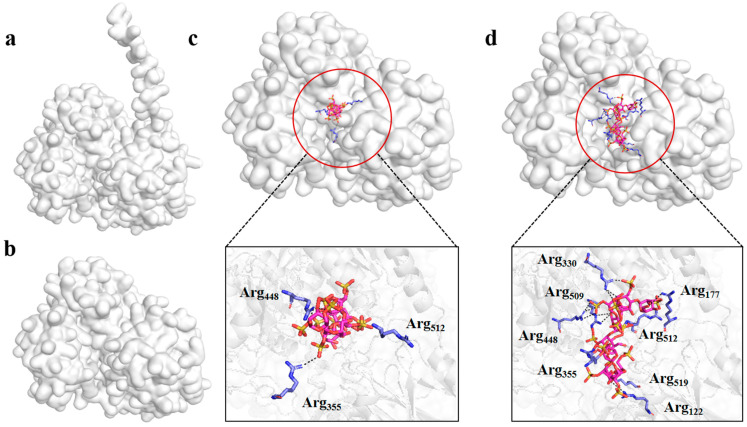
(**a**) OUC-CglA structure predicted by AlphaFold. A PQQ-like domain rich in β-pleated sheet is highlighted in red. (**b**) OUC-CglA-DPQQ structure predicted by AlphaFold. Molecular-docking profiles of OUC-CglA-DPQQ to Nλ6 (**c**) and Nλ8 (**d**). The models of ligands of Nλ6 and Nλ8 are shown, and the catalytic residues located in the catalytic pocket are illustrated in sticks modes.

Since the 3D structure of OUC-CglA-DPQQ was not available, it was constructed by AlphaFold2. It can be observed from Figure 5 that the protein structure of the mutant had a difference from that of OUC-CglA, and the PQQ-like domain at the N-terminal side was lost (Figure 5a,b). Molecular-docking results indicated that the ligands in Figure 5c,d both bound to the catalytic cave, and more arginine can be seen in the binding site of Nλ8 than that in Nλ6 (Figure 5c,d) [20]. As mentioned previously, arginine plays an extremely important role in other carrageenases’ binding sites [4,43,44,45]. It was found that Arg448, Arg512, and Arg355 were located in the catalytic groove, with a distance to Nλ6 of 1.9 Å, 2.8 Å and 3.3 Å, respectively. More interactions were formed between the substrate Nλ8 and arginine, Arg330, Arg509, Arg448, Arg355, Arg177, Arg512, Arg519 and Arg122, were located in the catalytic groove with a distance of 3.2 Å, 3.4 Å, 3.4 Å, 3.0 Å, 3.6 Å, 2.9 Å, 1.9 Å and 3.3 Å, respectively (Figure 5c,d), suggesting that the interactions between the arginine and the smaller substrate Nλ6 were weaker. Moreover, the results of the DoGSiteScorer revealed that the catalytic groove of OUC-CglA-DPQQ had a volume of 1380.83 Å^3^ and a surface of 1558.55 Å^2^, which were much bigger than those of OUC-CglA (885.18 Å^3^ and 920.75 Å^2^, respectively) [20]. This conformational change leads directly to an enlargement of the substrate binding site, and the enlargement of the substrate binding site made the mutant tend to bind the substrates with higher molecular mass and the binding force to the smaller substrates was weaker, explaining the reason for the mutant tending to produce long-chain oligosaccharides. The study of the mutant with the deletion of the PQQ-like domain has clarified that the PQQ-like domain is required for the production of short-chain λ-neocarrageenan oligosaccharides, and deleting the PQQ-like domain may be a promising method to produce long-chain λ-neocarrageenan oligosaccharides.

## 4. Conclusions

In summary, in this paper, a PQQ-like domain-deleted mutant OUC-CglA-DPQQ was constructed and characterized. Unlike other λ-carrageenases, the final products of the mutant OUC-CglA-DPQQ were long-chain λ-neocarrageenan oligosaccharides of Nλ10, Nλ12, Nλ14, Nλ16, Nλ18 and Nλ20, and the enzymatic properties changed a lot compared to its parental enzyme. Moreover, the deletion of the PQQ-like domain was also shown to increase the yield of λ-carrageenase by five-fold by enhancing the amount of enzyme in the lysate while decreasing the amount of inclusion bodies. Finally, since no enzymatic method of producing long-chain λ-neocarrageenan oligosaccharides has been reported so far, the λ-carrageenase mutant OUC-CglA-DPQQ has the potential to be a enzymatic tool for producing long-chain λ-neocarrageenan oligosaccharides.

## Figures and Tables

**Table 1:** Specific activity, molecular mass and protein content of the mutant OUC-CglA-DPQQ compared with OUC-CglA.

Protein	Total Protein (mg)	Pure Enzyme (mg)	Pure Enzyme Ratio (%)	Specific Activity (U/mg)	Molecular Mass (kDa)
OUC-CglA-DPQQ	84.438	3.28	3.9	51.59	93.7
OUC-CglA	59.248	0.68	1.1	418.68	105.1

## Data Availability

The original contributions presented in the study are included in the article/Supplementary Material, further inquiries can be directed to the corresponding author.

## Supplementary Materials

The following supporting information can be downloaded at https://www.mdpi.com/article/10.3390/foods13121923/s1, Table S1: Summary of the purification procedures for OUC-CglA-DPQQ.
