# Supplementary material for: Characterization of a λ-Carrageenase Mutant with the Generation of Long-Chain λ-Neocarrageenan Oligosaccharides
Source: Foods. 2024 Jun 18;13(12):1923. doi: 10.3390/foods13121923 (PMC11202985; doi:10.3390/foods13121923)
Supplement: Supplementary file 1 [file foods-13-01923-s001.zip › foods-3007520-supplementary.pdf]

Characterization of a λ-Carrageenase Mutant with the Generation of Long-Chain λ-Neocarrageenan Oligosaccharides

Zewei Lu [1], Hong Jiang [1,2,3,4,5,*], Dianqi Yang [6], Hengxin Tang [1], Hamed I. Hamouda [1], Tao Wang [5] and Xiangzhao Mao [1,2,3,4,5]

[1] State Key Laboratory of Marine Food Processing and Safety Control, College of Food Science and Engineering, Ocean University of China, Qingdao 266404, China
[2] Laboratory for Marine Drugs and Bioproducts of Qingdao National Laboratory for Marine Science and Technology, Qingdao 266237, China
[3] Qingdao Key Laboratory of Food Biotechnology, Qingdao 266404, China
[4] Key Laboratory of Biological Processing of Aquatic Products, China National Light Industry, Qingdao 266404, China
[5] Sanya Ocean Institute, Ocean University of China, Sanya 572024, China
[6] Department of Food Science and Technology, School of Agriculture and Biology, Shanghai Jiao Tong University, Shanghai 200240, China
* Correspondence: jh@ouc.edu.cn; Tel.: +86-532-82031360

Table S1. Summary of the purification procedures of OUC-CglA-DPQQ.

Purification steps	Total activity (U)	Total protein (mg)	Specific activity (U/mg)	Purification fold	Yield (%)
Crude enzyme	1888.25	83.44	22.63	1	100
Ni-NTA Superflow Purification	169.22	3.28	51.59	2.28	8.96
